# Brain Tumor Classification Using Meta-Heuristic Optimized Convolutional Neural Networks

**DOI:** 10.3390/jpm13020181

**Published:** 2023-01-20

**Authors:** Sarah Zuhair Kurdi, Mohammed Hasan Ali, Mustafa Musa Jaber, Tanzila Saba, Amjad Rehman, Robertas Damaševičius

**Affiliations:** 1 Medical College, Kufa University, Al.Najaf Teaching Hospital M.B.ch.B/F.I.C.M Neurosurgery, Baghdad 54001, Iraq; 2 Computer Techniques Engineering Department, Faculty of Information Technology, Imam Ja’afar Al-Sadiq University, Baghdad 10021, Iraq; 3 College of Computer Science and Mathematics, University of Kufa, Najaf 540011, Iraq; 4 Department of Medical Instruments Engineering Techniques, Dijlah University College, Baghdad 00964, Iraq; 5 Department of Medical Instruments Engineering Techniques, Al-Turath University College, Baghdad 10021, Iraq; 6 Artificial Intelligence & Data Analytics Lab, CCIS Prince Sultan University, Riyadh 11586, Saudi Arabia; 7 Faculty of Applied Mathematics, Silesian University of Technology, 44-100 Gliwice, Poland

**Keywords:** brain tumor, MRI, Harris Hawks optimization, deep learning, healthcare, health risks

## Abstract

The field of medical image processing plays a significant role in brain tumor classification. The survival rate of patients can be increased by diagnosing the tumor at an early stage. Several automatic systems have been developed to perform the tumor recognition process. However, the existing systems could be more efficient in identifying the exact tumor region and hidden edge details with minimum computation complexity. The Harris Hawks optimized convolution network (HHOCNN) is used in this work to resolve these issues. The brain magnetic resonance (MR) images are pre-processed, and the noisy pixels are eliminated to minimize the false tumor recognition rate. Then, the candidate region process is applied to identify the tumor region. The candidate region method investigates the boundary regions with the help of the line segments concept, which reduces the loss of hidden edge details. Various features are extracted from the segmented region, which is classified by applying a convolutional neural network (CNN). The CNN computes the exact region of the tumor with fault tolerance. The proposed HHOCNN system was implemented using MATLAB, and performance was evaluated using pixel accuracy, error rate, accuracy, specificity, and sensitivity metrics. The nature-inspired Harris Hawks optimization algorithm minimizes the misclassification error rate and improves the overall tumor recognition accuracy to 98% achieved on the Kaggle dataset.

## 1. Introduction

The role of clinical diagnosis in modern healthcare has increased in significance. Medical imaging researchers have focused extensively on brain cancer because it is the deadliest disease in the world [1]. A brain tumor is one of the uncommon growths of the cell in the brain. Most tumors are benign, and few are caused by cancerous or malignant cells [2]. Brain tumors that originate in the brain are known as primary brain tumors. Secondary brain cancer or brain metastases refers to cancer that has spread from another part of the body to the brain [3]. Brain tumor symptoms vary according to the tumor size and part of the brain involved, such as difficulties in sensations, speaking, walking, mental changes, vomiting, vision, seizures, and headaches [4]. Brain tumors are difficult to diagnose since their clinical presentations vary widely based on factors such as tumor type, location, size, and growth rate. Regarding pathogenesis, the clinical manifestations may be classified as either increased intracranial pressure or localized, indicating damage to native tissue. Multiple primary symptoms are present in the clinical presentation of numerous patients, for example, headache and altered mental status; therefore, the total rate is more than 100%.

An early and precise diagnosis based on magnetic resonance imaging (MRI) and computed tomography (CT) can improve brain tumors’ medical examination and prognosis. Afterward, the biopsy test confirms the disease, and the tumor’s severity is identified from the findings. The American Brain Tumour Association (ABTA) and World Health Organization (WHO) report clearly state that brain tumors are divided from grade I to grade IV scale [5]. The division is used to classify malignant and benign tumors. Benign tumors come under grades I & II, whereas malignant tumors belong to grade III, and glioma falls under grade IV [6]. Grades I and II tumors have slow growth, the low-grade tumor type. Grades III and IV tumors have rapid growth and are named malignant brain tumors [7]. If people are affected by grade II tumors, they require continuous monitoring and observation. Every 6 to 12 months, patients must take computed tomography (CT) or magnetic resonance imaging (MRI) tests to monitor their health status. Experts process the radiographic images to identify the normal and abnormal tissues to determine the tumor grade level. The captured MRI [8,9] is utilized to investigate the tissues such as white matter (WM), cerebrospinal fluid (CSF), and gray matter (GM) by applying various feature extraction and classifier approaches. These tissues require segmentation procedures [10] to identify the various issues. The MRI has various modalities that require a continuous examination to identify tumor tissues, such as dead and edema [11,12]. The tumors are detected early, so proper treatment is initiated to reduce risk factors such as age, gender, home and work exposures, family history, and ionizing radiation.

Therefore, the MRI segmentation process [12] is applied in this work to investigate image modalities. Image properties such as texture, color, boundaries, and contrast details are computed during the segmentation to improve the tumor detection rate. Image segmentation uses several methods to identify disease-affected regions with minimum computation complexity. This process uses the active contour method [13] to solve the intensity homogeneity issues. Various statistical and textural features are extracted from the segmented regions to identify the brain tumor. Several classifiers [14], such as fuzzy clustering means (FCM), Artificial Neural Networks (ANN), Support Vector Machine (SVM), Expectation-Maximization (EM), and knowledge-based techniques are introduced to recognize brain tumors [15,16]. However, it is difficult to identify the exact tumor region and hidden edge details with minimum computational complexity [17,18]. 

Radiologists can benefit from computer-aided diagnosis techniques if they can properly detect brain tumors in medical images by detecting, segmenting, and classifying them. However, radiologists find the manual process of detecting brain tumors tedious and prone to error. Existing methods are complex in identifying the exact tumor regions and boundaries, which causes to minimization of overall recognition accuracy. In addition, the limited availability of the features creates the maximum classification error rate. Therefore, strength training patterns are required to improve the testing and training feature matching [19,20].

Furthermore, the classification problem causes reliability and scalability issues for MRI-based brain tumor identification. To overcome these problems, we propose the Harris Hawks optimized convolutional neural network (HHOCNN) method. The method uses the Harris Hawks Optimization algorithm [21], which has been successfully used in several domains, such as machine scheduling [22], speech recognition [23], and disease recognition [24]. HHO is a population-based optimization technology consisting of three stages: exploration, transformation, and exploitation. The potential application of a novel HHO metaheuristic algorithm for multilevel image thresholding helps to classify tumors. This swarm-based algorithm was designed to handle continuous optimization tasks and produce high-quality solutions efficiently. A primary motivation for this paper is to determine the optimum thresholds for image classification and segmentation based on the hybridization of the HHO and differential evolution algorithm.

Initially, brain MRIs are collected from the Kaggle dataset to analyze them. Image enhancement and denoising tools are used to strengthen the image and remove noise (duplicates) to minimize the false tumor recognition rate. The candidate region method investigates the boundary regions with the help of the line segments concept, which reduces the loss of hidden edge details. Different features are extracted from the segmented region, which is classified by applying the proposed HHOCNN model. The convolutional neural network (CNN) computes the exact region of the tumor with fault tolerance. 

The main contribution of this study is listed as follows.

- Edge loss minimization while segmenting tumor region by using candidate region from MRI.
- Use of an optimization algorithm that updates the network parameters and normalizes the classification process.
- Maximizing brain tumor detection rate using the optimized segmentation process and classification algorithm.

The paper is organized as follows. Section 2 analyzes the various researchers’ works on the brain tumor identification process. Section 3 discusses the Harris Hawks optimized convolutional network-based brain tumor detection process. Finally, Section 4 evaluates the system’s effectiveness, and the conclusion is discussed in Section 5.

## 2. Related Work

When other methods fail to yield desirable results, turning to a neural network to solve a complex regression problem is effective. An effective neural network model is discovered by exploring a high-dimensional hyper-parameter and weight space in search of the points of convergence. The advantages of a refined evolutionary competitive algorithm and gradient-based backpropagation have recently been combined in several frameworks that integrate neural networks with heuristic (or nature-inspired) optimization algorithms [25].

Irmak et al. [26] proposed deep convolution with a fully optimized framework (DC-FOF) to identify brain tumors from MRIs. The main intention of this system is to resolve the multi-classification problem by applying CNN. The network classifies the tumors into metastatic, pituitary, meningioma, glioma, and normal regions. The fully convoluted network classifies the tumor grade with around 98.14% accuracy. During the analysis, grid search optimization techniques are utilized to fine-tune parameters, reducing the misclassification error rate. The effectiveness of the introduced system is evaluated using InceptionV3, AlexNet, GoogleNet, VGG-16, and ResNet-50.

Rehman et al. [27] detected brain tumors from MRIs using 3D CNNs and a feature selection process. The CNN uses the fully connected layer to extract the region. The identified regions are transferred to the pre-trained network that extracts the various features. The correlation between the features is investigated to select the best features. Finally, the derived features are validated using feed-forward neural networks that recognize the brain tumor with minimum complexity and high accuracy.

Garg et al. [28] proposed hybrid ensemble classifiers (HEC) for detecting brain tumors from MRIs. This work aims to resolve the difficulties involved in the boundary, volume, size, and shape detection process. First, the collected MRIs are processed by the Otsu threshold that extracts the affected region. Then, various methods such as the gray level co-occurrence matrix, the principal component analysis (PCA), and the stationary wavelet transform (SWT) are applied to derive the features. Finally, the extracted features are processed by hybrid ensemble techniques (decision tree, k-nearest neighboring, and random forest) which recognize the tumor region according to the majority voting method. The hybrid classification technique consumes less computation time compared to other methods. 

Addeh et al. [29] detected brain tumors using deep features and optimized radial basis function neural networks (RBFNN). This work uses the brain tumor segmentation (BraTS) 2015 dataset. The images are investigated using the grab-cut approach that identifies the tumor-affected region. Then CNN is applied to extract the deep features. The features are passed to the radial function network that classifies the tumor with the help of the effective training process. The network uses the bee’s algorithm to learn, improving the overall classification accuracy. The optimized method-based learning techniques simplify the classification procedure and error rate. 

Islam et al. [30] detected brain tumors from MRIs using a template-based K-means clustering algorithm (TK). This system’s main intention is to recognize brain tumors with minimum execution time. First, the MRI is investigated using a superpixel and a PCA approach that derives valuable information. Then the TK approach is applied to segment the region, which helps to identify the brain tumor region with minimum time and maximum accuracy. 

Zhang et al. [31] segmented brain tumor regions using the multi-encoder net framework (MENF). The main intention of this system is to reduce the difficulties present in the feature extraction process and to reduce loss value. Initially, 3D brain images are collected from the BraTs2020 dataset, which is processed by multiple encoders. The encoder technique uses a set of weights for updating the network parameters, which helps reduce the voxel imbalance problem. Furthermore, this process ensures high tumor recognition accuracy with a minimum error rate. 

Munir et al. [32] applied a 2D-UNET CNN for segmenting brain tumors. This system intends to minimize hurdles in spatial variability and large structural deviations. The images are collected from the BraTS 2019 dataset, which is processed by CNNs that recognize brain tumors effectively. As a result, the introduced system achieves the Dice coefficient of 0.9694. 

Biratu et al. [33] segmented brain tumor regions from an MRI using an enhanced region-growing approach. This study uses the BraTS2015 dataset for analyzing brain tumor regions. The collected images are processed using the thresholding approach that divides the image region into eight blocks that estimate the region intensities. Then, the region-growing algorithm is applied to identify the tumor-affected region. From the region, a deep learning algorithm is used to recognize the tumor with 90% accuracy. 

Chahal et al. [34] applied the hybrid weighted fuzzy approach to identify the brain tumor region from an MRI. The images are collected from the DICOM dataset, a processed fuzzy clustering approach that segments the region according to the fuzzy membership function. The fuzzification process helps to group similar pixels with minimum computation complexity. The affected region is classified using a SVM that classifies the tumor with 97% accuracy. 

Maqsood et al. [35] proposed a method for the detection of brain tumors that relies on fuzzy logic edge detection and the U-NET classifier. The proposed system for tumor segmentation employs fuzzy logic in conjunction with image enhancement, edge detection, and classification. The input images are pre-processed with a contrast enhancement technique, the source images’ edges are identified with a fuzzy logic-based edge detection technique, and various scale levels of dual tree-complex wavelet transform (DTCWT) are employed. Features are computed from decayed sub-band images and then classified with the U-NET CNN classifier, which can tell the difference between meningioma and non-meningioma brain tumors.

Maqsood et al. [36] proposed a five-step procedure for the detection and classification of brain tumors. First, the source image’s edges are identified using linear contrast stretching. Then, the brain tumors are segmented, utilizing a 17-layered neural network architecture. Finally, the extracted features are analysed using a variant of the MobileNetV2 architecture. For classification, the study used a multiclass SVM with a method that controls entropy to select features (M-SVM). It enables distinguishing between meningioma, glioma, and pituitary tumors in images using the M-SVM, which is used for brain tumor classification. With an accuracy of 97.47% and 98.92% on the BraTS 2018 and Figshare datasets, respectively, the proposed method outperformed other methods.

Rajinikanth et al. [37] investigated the performance of pre-trained VGG16 and VGG19 schemes in detecting the grade of a brain tumor (glioma/glioblastoma) using various pooling methods. SoftMax is used to perform the classification. The images of brain tumors were obtained from The Cancer Imaging Archive (TCIA). The experimental results show that the VGG16 with average pooling outperforms other methods in terms of classification accuracy (>99%) with Decision Tree (DT).

Rajinikanth et al. [19] performed brain tumor segmentation using VGG-UNet, and deep-feature extraction using the VGG16 network. The extracted features were fused with handcrafted features and the best features were selected using the firefly-algorithm. The classification was done using the SVM-Cubic and an accuracy of 98% was achieved.

In [38], AlexNet’s convolutional neural network (CNN) was used to conduct brain MRI classification for brain tumor detection, obtaining an overall accuracy of 99.62%.

According to various researchers’ studies, brain tumor is recognized by various image processing and machine learning techniques. The existing methods are utilized to resolve computation difficulties while investigating pixels in MRI. However, most of the time, edge inner details are difficult to identify, which causes an increase in the difficulties of exact tumor location segmentation. This problem increases the misclassification error rate and classification problem. To overcome these issues, meta-heuristic optimized convolution neural networks are applied in this work to reduce the difficulties in brain tumor segmentation. 

## 3. Meta-Heuristic Optimized Convolution Neural Network for Brain Tumor Detection

### 3.1. Workflow

**Figure 1:**
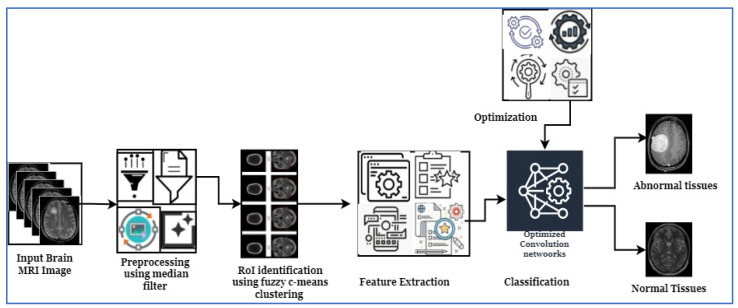
Proposed workflow of brain tumor classification.

This section discusses the methodologies applied to perform the brain tumor classification using a nature-inspired metaheuristic optimized CNN. The process contains the following stages: image collection, image noise reduction, image segmentation and clustering, feature extraction selection, and then classification. Figure 1 illustrates the brain tumor classification from MRI processing using the Harris Hawks optimized CNN (HHOCNN). The definition of gross tumor volume is one of the most crucial steps in preparing radiotherapy treatment. This step is the foundation for both the design and delivery of treatment. If the applied safety margins are insufficient to compensate for an error in tumor delineation, a systematic geographical miss may occur during treatment administration. This could diminish the likelihood of tumor control. Inadequate 3D target definition may prevent the full potential of advanced treatment planning techniques from being realized, or result in a loss of tumor control due to geographic dose misses. This study summarizes and discusses the most relevant issues when utilizing MRI for target volume delineation in intracranial stereotactic radiotherapy. This study aims to reduce the misclassification error rate and improve overall tumor recognition accuracy. Here, the effective segmentation technique is applied to identify the region of interest because it is used to derive the tumor-related features effectively. Then the detailed working process of introduced brain tumor recognition is illustrated in Figure 1.

Figure 1 illustrates the brain tumor classification working structure. Here, the MRI brain images are collected from the Kaggle dataset, which is processed by the median filter to remove the noise. After that, the fuzzy c-means clustering algorithm and region of interest are applied to identify the candidate region. Then various features are extracted from the segmented region to identify the normal tissue (non-cancerous) and abnormal tissue (cancerous) from the MRI. The detailed working process of the MRI-based brain tumor recognition process is discussed in the following section.

### 3.2. Dataset

**Figure 2:**
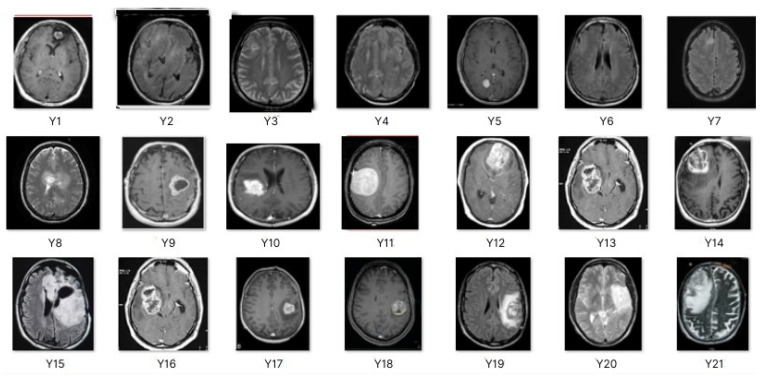
MRI samples Normal Brain MRI (Y1 to Y8) Benign tumor MRI (Y9 to Y15) Malignant tumor MRI (Y16 to Y21).

This work collects images from the Kaggle Brain MRI for Brain Tumor Detection dataset, which consists of 253 files with cancer and non-cancer brain images. The sample MRI is illustrated in Figure 2. The dataset has two folders: no tumor encoded as 0 and tumor as 1. The labeled data is more helpful in extracting the patterns from the MRI, which is used to predict the new images related output. The collected image consists of noise information that reduces the performance of brain tumor recognition accuracy. Therefore, image noise should be eliminated to improve the overall prediction efficiency.

### 3.3. Pre-Processing

The first step of this study is to eliminate noise from the brain MRI because it affects the entire tumor region identification. Then, the collected images are investigated according to the contrast and pixel information to maximize the MRI quality. During this process, low-level information is identified and eliminated at an earlier stage. Here, the pixel intensities are examined to maximize the image quality. Therefore, this work applies a median filter and histogram equalization to enhance the overall MRI quality. First, the images are decomposed into sub-images to analyze quality and quantity. Then, every pixel is compared with the threshold value; if the pixel is corrupted by noise, it has been replaced by the median value. During this process, neighboring pixels are arranged in a sorting order, and the median value is selected to replace the noise pixel value. After that, contrast of the image is improved by using histogram equalization. 

The normalized Median Filter (MF) is described as follows:

(1) $$f\left(j,i\right)=Median\left\{g\left(o,t\right),\left(o,t\right)\in S_{ji}\right\}$$

As shown in Equation (1), where g(o,t) denotes the noise, the median filtering method is to sort the pixels in the sliding filter window and the output pixel value f(j,i) of the filtering; the result is the median value of the series.

Consider that the data set has S samples with a gray level of pixel information Gr, which is denoted as $P_{0},P_{1},\ldots .P_{Gr-1}$. For every pixel, intensity and cumulative distribution value should be computed to identify the quality of the pixel. Then the pixel quality is determined according to the density function defined in Equation (2).

(2) $$PDF\left(P_{n}\right)=\frac{n^{h}}{n}$$

Here h=0,1,2,3,…..Gr−1, brain image pixel value is denoted as $n^{h}$, n is the number of pixels in S. 

The weighted mean value of the pixel is computed to identify the cumulative distribution of the pixel in Equation (3).

(3) $$X_{t}=\frac{\sum_{lg=a}^{b}l*CDF\left(lg\right)}{\sum_{lg=a}^{b}CDF\left(lg\right)}$$

Here an image gray pixel value is defined as lg, the sub-interval histogram value is defined as (a, b), which generally belongs to (0–255), t is the interval value (0≤r≤t−1), and r is the sub histogram $\left(X_{r},X_{r+1}\right)$. After that, the sub-images should be identified to calculate the image histogram.

(4) $$S_{k}=\left\{S\left(x,y\right)|X_{k}\leq S\left(x,y\right)X_{k+1},\forall S\left(x,y\right)\in S\right\}$$

Here sub-images of S are represented as $S_{k}$ where k=0,1,2,…..t−1. 

The probability and cumulative distribution of gray-level images are computed:

(5) $$PDF_{k}\left(P_{h}\right)=\frac{n^{k}}{n_{k}}$$

(6) $$CDF\left(Gr_{j}\right)=\overset{n}{\underset{j=X_{0}+1}{\sum }}PDF_{k}\left(Gr_{j}\right)$$

here k=0,1,2,….t−1 and $h=X_{k}+1,X_{k}+2,\ldots X_{k+1}$ and the probability distribution of the pixels is represented as $PDF_{k}\left(P_{h}\right)$ which uses the entire pixels in the image. 

Histogram equalization is a technique for processing images that modifies the histogram’s distribution of intensities to improve an image’s contrast. Histogram equalization is performed according to the computed mean function and piecewise details. This process performed for every sub-image that successfully eliminates unwanted information also improves the image quality. The noise removed and histogram-enhanced images are illustrated in Table 1. The brain MRI pre-processed output images are illustrated in Table 1, which has sample brain MRI, noise-removed images, image histogram analysis, and contrast enhancement images. The MRI images of brain tumors are of a higher quality and more suitable for further processing by clinical specialists or imaging modalities after the pre-processing stage. These images state how effectively the introduced system removes noise from the MRI. Then the tumor-affected region should be predicted by applying the segmentation technique discussed below.

### 3.4. Segmentation

The second step of this study is identifying the tumor region from the pre-processed MRI. Brain tumor detection is greatly affected by the unclear and uncertain boundary details presented in the MRI. The boundary uncertainty issue causes computation complexity and affects the segmentation accuracy. To resolve these issues, the fuzzy c-means clustering (FCM) approach is used in this work. 

FCM uses the fuzzy set and membership values to minimize uncertainty. First, the degree of membership value is applied to resolve the uncertainty problem because it helps to identify the relationship between each pixel. The computed pixel relationship is used to determine the cluster center value. Then the cluster center is defined by Equation (7).

(7) $$V_{j}=\frac{\left(\sum_{i=n}^{n}{\left(\mu_{ij}\right)}^{m}x_{i}\right)}{\left(\sum_{i=n}^{n}{\left(\mu_{ij}\right)}^{m}\right)},\forall j-1,2,\ldots .c$$

Here n is the number of pixels in the image, $V_{j}$ is represented as the *j*-th cluster center, the fuzziness index value is denoted as m; m∈(1,∞), the number of cluster centers is defined as c, and the fuzzy membership value for *i*-th data’s *j*-th cluster center is defined as $\mu_{ij}$. 

The fuzzy membership value $s_{i}$ is computed from the Euclidean distance $d_{ij}$ between the *i*-th data *j*-th cluster center. Then the fuzzy membership values are calculated:

(8) $$\mu_{ij}=\frac{1}{\sum_{k=1}^{c}{\left(d_{ij}/d_{ik}\right)}^{\left(2/m-1\right)}}$$

Therefore, the values for every data point $X=\left(x_{1},x_{2},\ldots .x_{n}\right)$ should be analyzed, and the set of centers $V=\left(v_{1},v_{2},\ldots .v_{c}\right)$ is computed to identify the tumor-affected region. Furthermore, the candidate region investigates the boundary region of the line segment, which reduces the loss of hidden edge details. 

The candidate region is a potentially engaging circle, and the one with the greatest number of line segments is selected. The best circle from the list of interesting circles is the one with the most line segments. The optimal region is the one containing the optimal circle. The candidate region is identified by selecting the seed points according to the pixel-wise analysis. The seed points are utilized to determine whether the neighboring pixels belong to the cluster. Here, the selected cluster center $V_{j}$ is treated as the seed point because it is determined according to the grey pixel criteria and other pixel characteristics such as texture, color, intensity, and membership criteria. 

The selected seed points are compared with the neighboring pixel value, and if it is matched with the threshold value, the pixels are grouped and form the cluster. This process was performed in an iterative manner and identified the affected region successfully. 

The segmentation process should be complete, which means it covers the entire $\cup_{i=1}^{n}R_{i}=R$ region. The region pixels are connected $R_{i};\;i=1,2,\ldots n$, which helps to identify the disjoint regions $R_{i}\cap R_{j}=\emptyset ,\;i\neq j$. The effective computation of each pixel is used to determine the same region pixel by checking the condition $P\left(R_{i}\right)=True;i=1,2,\ldots .n$. Here, $P\left(R_{i}\right)$ is defined as the logical predictor of the image pixel used to identify the same grayscale pixels. If the condition is $P\left(R_{i}\cup R_{j}\right)=False$ for any adjacent pixel region $R_{i}$ and $R_{j}$ is discarded, and the searching process is continued to cover the entire pixel in S. This process is repeated until it reaches the $U^{\left(k+1\right)}-U^{\left(k\right)}<\beta$. Here, termination criteria are defined as β, which has a (0/1) value. 

This process covers the image pixels and resolves the uncertainty issues while segmenting the affected region. The effective computation of pixel information helps preserve the edge information, reducing the complexity involved in the inner details processing. According to the discussion, the segmented regions are illustrated in Table 2.

The segmented region and respective edge-related area are illustrated in Table 2. The segmented regions are processed by feature extraction to derive the various information from MRI images. The effective utilization of seed points helps to identify the candidate region successfully, improving the overall tumor recognition accuracy. 

### 3.5. Feature Extraction

An edge detection approach can recognize a tumor’s shape and location in an MRI using feature extraction based on approximate reasoning. Texture feature extraction can be described as a statistical technique that discloses specific properties about the spatial distribution of gray levels in image texture, considering the spatial connection of pixels. The tumor and normal regions have specific textures and spectral information that differentiate normal and abnormal tissue growth. Therefore, each feature should be retrieved, such as standard deviation, mean, moment skewness, kurtosis, and other statistical information. This information is highly useful to identify whether the segmented regions belong in the cancerous or non-cancerous category. 

Then the source code for feature extraction is given in Appendix A.

### 3.6. Cancer Classification

The last stage of this study is classifying the brain tumor by applying the meta-heuristic optimized CNN. The CNN model has input, convolution, a rectified linear unit, a pooling layer, and a fully connected layer. These layers are more useful for investigating every small region in a segmented image. First, the images are processed by a convolution layer that decomposes the images into a small region. Then, the ReLu process uses the element-wise activation function to operate, and the pooling is applied to minimize the dimensionality of the feature set. Finally, the output is obtained in the fully connected layer, which gives labels 1 and 0 to the input. 

The collected images are divided into training and testing phases. Initially, the training phase uses the above-discussed steps, and the output patterns are generated, which are used to minimize the loss function deviations. The computation complexity, i.e., time and cost, are minimized according to the pre-training model. In the pre-training process, images automatically extract the information using different layers, and the obtained outputs are stored in the database. Then, the testing images are matched with the trained patterns to reduce the loss function. Here, the gradient descent algorithm is utilized to measure the loss function. If the system ensures high deviations, then the network parameters must be updated, and learning should be propagated. 

The Harris Hawks optimization algorithm is utilized in this work to perform the network parameter regularization process. First, the solution has to be initialized; the number of solutions is defined as f, and the position of the solution is $A_{e}$ for eth solution. Therefore, the solution position is represented as $A=\left\{A_{1},A_{2},\ldots ..A_{f}\right\}$. Before updating the network parameter, a deviation, the mean square error (MSE) value, should be calculated (Equation (9)).

(9) $$MSE_{error}=\frac{1}{g}\sum_{e=1}^{g}{\left[F_{g}-F_{g}{}^{*}\right]}^{2}$$

Here the deviation between the $F_{g}$ expected and predicted $F_{g}{}^{*}$ output is measured using the deviation error rate $MSE_{error}$. The deviations are computed for several samples g, 1<e≤g. Suppose the network produces the error rate; network parameters should be updated according to the optimal global solution. The optimization algorithm needs a minimum parameter to fine-tune the network operations. The updating solution fine-tunes the network parameter according to Equation (10).

(10) $$A\left(b+1\right)=A_{rand}+BC$$

here $A_{rand}$ is denoted as a random number, coefficient vectors are denoted as B, and the whale-prey distance is defined as C. Then the optimization problem is solved by updating the following equations.

(11) $$A\left(b+1\right)=A_{rand}-v1\left|A_{rand}-2v2\;A\left(b\right)\right|$$

Assume, $A_{rand}\geq 0.5,$ then the parameter updating procedure is defined in Equations (12) and (13).

(12) $$A\left(b+1\right)=A_{rand}-v1A_{rand}+2v1v2A\left(b\right)$$

(13) $$A_{rand}=\left[\frac{1}{\left(1-v1\right)}\left[A\left(b+1\right)-2v1v2A\left(b\right)\right]\right]-BC$$

This process produces several global solutions that are used to update the network parameters, and the updating equations are defined in Equation (14).

(14) $$A\left(b+1\right)=\frac{1-v1}{v1}\left(\frac{2v1v2A\left(b\right)}{\left(1-v1\right)}+BC\right)A\left(b+1\right)a$$

The computed network parameters balance the exploitation and exploration problems with a minimum error rate. Then the convergence rate should increase by selecting the optimal global solution. The error rate is again calculated to recompute network parameters and improve the system’s performance. With the help of the updated parameters, the network computes the output values as follows:

(15) $$S_{f}^{u}=Z\left(a_{f}^{u}\right);\;Z\left(a_{f}^{u}\right)=\overset{W_{1}{}^{p-1}}{\underset{p=1}{\sum }}\overset{W_{2}{}^{p-1}}{\underset{m=1}{\sum }}\overset{W_{1}{}^{p-1}}{\underset{n=1}{\sum }}\left(V_{f,p,m,n}^{u}\right){\left(T_{f}^{u-1}\right)}_{m.n}$$

Here the weight of layer (*u* − 1) is denoted as $V_{f,p,m,n}^{u}$. These weight values are continuously updated to reduce the error rate. Here, $T_{f}^{u-1}$ estimated using an activation function defined as $T_{f}^{u}=fun\left(T_{f}^{u-1}\right)$. Then the classification source code is defined as follows.

The source code for classification is given in Appendix B.

Thus, the optimization algorithm effectively reduces computation complexity and deviation between the actual and predicted values. Moreover, the minimum classification complexity increases the overall recognition accuracy. Then the effectiveness of the system is evaluated as shown in the section below. 

## 4. Results and Discussion

This section evaluates the excellence of the introduced meta-heuristics optimized convolution neural network (HHOCNN) based brain tumor recognition from the MRI. In this work, the Kaggle brain tumor dataset consists of 250 brain images with “yes” and “no” folders. The labeled information is more helpful in classifying the testing images with maximum accuracy. The discussed system is implemented using MATLAB tool, and the defined image processing and machine learning techniques are applied to recognize the tumor. In this work, the weighted median filter is applied to remove the noise from the MRI, which is more helpful in eliminating irrelevant and inconsistent details. The noise removal process reduces the computation complexity and difficulties in region segmentation.

Further, fuzzy membership-based seed points are more practical for recognizing the candidate region. The introduced method investigates every region, which maximizes the inner details examination procedure. The effective utilization of segmentation procedures improves the overall tumor recognition accuracy. Traditional accuracy, specificity, precision, recall, and F1-score metrics are utilized to determine the system’s efficiency. 

The system’s effectiveness is evaluated using the prescribed performance metrics, and the results are shown in Table 3. The introduced system efficiency is compared with deep convolution with a fully optimized framework (DC-FOF) [16], hybrid ensemble classifiers (HEC) [18], optimized radial basis function neural networks (RBFNN) [19], and multi-encoder net framework (MENF) [21]. The existing algorithm works effectively while recognizing the tumors. These methods utilize the hybridized function and segmentation procedures that minimize the computation complexity in the beginning stage. For these reasons, in this work, [16,18,19,21] are utilized for comparison purposes. The Confusion Matrix is a useful machine learning technique that measures recall, precision, accuracy, and the AUC-ROC curve. The ROC curve is utilized to evaluate a test’s total diagnostic performance and compare the performance of two or more diagnostic tests. It is utilized to choose optimal cut-off values for identifying the presence or absence of a brain tumor.

**Figure 3:**
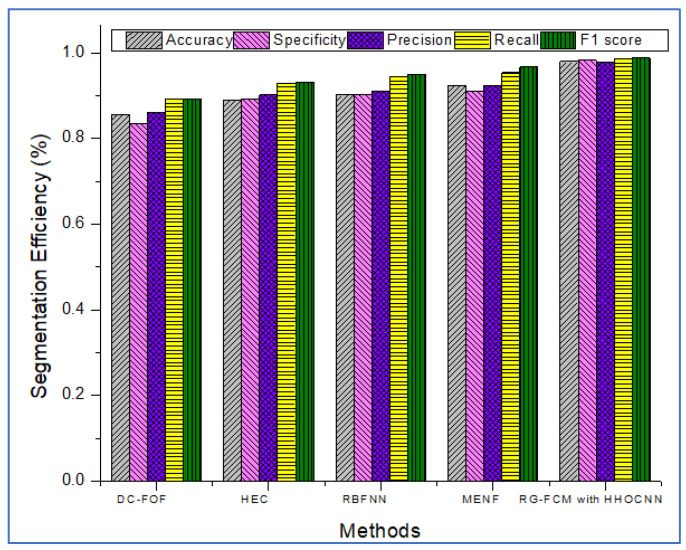
Segmentation Efficiency.

Table 3 also demonstrates that introduced region growing with fuzzy C-means clustering (RG-FCM) with the HHOCNN approach ensures high brain segmentation accuracy. The obtained results are maximum compared to the existing approach. Here, the RG-FCM with HHOCNN approach uses the seed points to select the cluster center. In addition, fuzzy membership functions are utilized to determine the exact center criteria. This process helps to reduce uncertainty issues while investigating the pixel regions effectively. Moreover, the CNN has different layers that also segment the region in the pre-training model. This process leads to minimizing the deviation between the actual and predicted values. Due to the effective analysis, the RG-FCM with HHOCNN approach attains 98%, as opposed to deep convolution with a fully optimized framework (DC-FOF) [16] (85.6%), hybrid ensemble classifiers (HEC) [18] (89%), optimized radial basis function neural networks (RBFNN) [19] (90.2%), and multi-encoder net framework (MENF) [21] (92.3%). According to the discussion, the graphical analysis of the introduced brain tumor identification process is illustrated in Figure 3.

The successful examination of each region $\cup_{i=1}^{n}R_{i}=R$, disjoint regions $R_{i}\cap R_{j}=\emptyset ,\;i\neq j$ helps identify the seed points effectively. From the identified seed points, each region pixel is compared with $P\left(R_{i}\right)=True$ and $P\left(R_{i}\cup R_{j}\right)=False$ conditions to identify the segmented region boundaries effectively. During this process, region seed points are determined according to the number of pixels in the image and the fuzziness index. These values from the cluster center criteria are defined as $\frac{\left(\sum_{i=n}^{n}{\left(\mu_{ij}\right)}^{m}x_{i}\right)}{\left(\sum_{i=n}^{n}{\left(\mu_{ij}\right)}^{m}\right)}$. 

Based on the center value, the remaining pixels are investigated successfully to determine the candidate region. The use of the fuzzy membership function $\frac{1}{\sum_{k=1}^{c}{\left(d_{ij}/d_{ik}\right)}^{\left(2/m-1\right)}}$ improves the exactness of the segmented region. The segmented region is more useful for recognizing the tumor and non-tumor cells with a minimum error rate. 

The introduced classifier has different layers that use the convolution layer to minimize the inconsistent data, and the weighted mean average method eliminates irrelevant details. This noise removal process increases the overall tumor recognition accuracy. Moreover, their HHO optimization algorithm updates the network parameters according to the whale food searching process. 

The network parameters are fine-tuned in every iteration using the $A\left(b+1\right)=\frac{1-v1}{v1}\left(\frac{2v1v2A\left(b\right)}{\left(1-v1\right)}+BC\right)A\left(b+1\right)$ value. The optimization process uses the coefficient vector and prey distance measure that helps to reduce the misclassification error rate and maximize the overall prediction accuracy.

The method uses the fully convolute layer functions $\overset{W_{1}{}^{p-1}}{\underset{p=1}{\sum }}\overset{W_{2}{}^{p-1}}{\underset{m=1}{\sum }}\overset{W_{1}{}^{p-1}}{\underset{n=1}{\sum }}\left(V_{f,p,m,n}^{u}\right){\left(T_{f}^{u-1}\right)}_{m.n}$ to recognize the outputs (normal and abnormal). The network parameters are continuously regularized according to the whale prey searching procedure. The effective utilization of each function, objective function, and iteration helps retrieve the testing-related features more accurately. The obtained results are higher compared to the existing methods, such as deep convolution with a fully optimized framework (DC-FOF) [16], hybrid ensemble classifiers (HEC) [18], optimized radial basis function neural networks (RBFNN) [19], and multi-encoder net framework (MENF) [21] (see Table 3 and Figure 3).

CNN uses the convolutional layer to sample the input image, reducing the involvement of noise pixels and almost reducing the misclassification error rate. Moreover, the proposed model uses the seeding point and fuzzification procedure to resolve uncertainty issues. These two issues minimize the computation complexity while investigating the MRI brain tumor patterns. The activation function and fully connected networks generate the training patterns that help to match the training patterns. 

The region growing with fuzzy C-means clustering (RG-FCM) with the HHOCNN approach attains maximum recognition accuracy. As stated, the computed output is compared with Equation (9) to compute the deviations. The deviation between the $F_{g}$ expected and predicted $F_{g}{}^{*}$ outputs is extremely low. If the computed values are 1<e≤g minimum, it has been back propagated again to estimate the output value. During this process, the network performance is regularized by updating the network parameter values. The updating procedure uses the whale prey searching process. Here, the parameters are updated according to the $\frac{1-v1}{v1}\left(\frac{2v1v2A\left(b\right)}{\left(1-v1\right)}+BC\right)A\left(b+1\right)$ value which improves the overall recognition accuracy. The obtained results exceed the performance of the existing reported approaches (see Table 3 and Figure 3).

## 5. Conclusions

This paper has presented a region growing-fuzzy c-means clustering model optimized with Harris Hawks CNN-based brain tumor recognition process from MRI. The proposed methodology is evaluated on the MRI collected from the Kaggle dataset, which consists of both normal and abnormal brain images.

The median filter processes the collected images to eliminate irrelevant and inconsistent details, reducing pixel handling difficulties. The tumor-affected regions are predicted by selecting the seed points according to the fuzzification procedure. The fuzzy set selects the number of cluster centers that identify the regions’ relationship. The fuzzy-based computation minimizes the uncertainty issues while segmenting the region. This process minimizes the complexity in edge details identification because of the effective search of the region. Finally, distinctive features are extracted that are classified using the convolute network. The fully convolute layer recognizes the output by fine-tuning the parameter according to the HHO algorithm. The nature-inspired Harris Hawks optimization algorithm minimizes the misclassification error rate and improves the overall tumor recognition accuracy.

In the future, this study will incorporate an optimized feature selection and learning model to improve brain tumor analysis.

## Figures and Tables

**Table 1:** Sample Preprocessing MRI.

MRI	Pre-Processed Images	Image Histogram	Contrast Improved Images
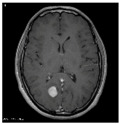	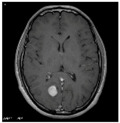	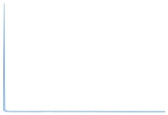	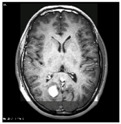
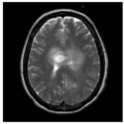	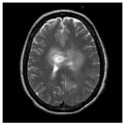	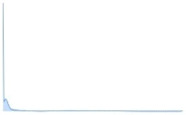	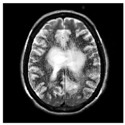
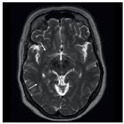	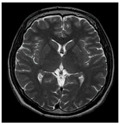	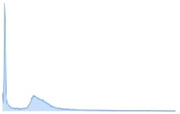	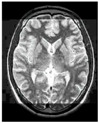
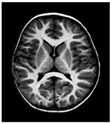	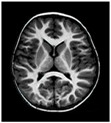	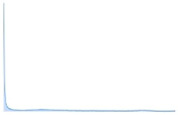	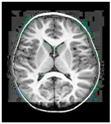

**Table 2:** Sample brain tumor region segmented images.

Enhanced Image	Edge Detected Image	Segmented Image	
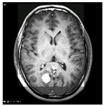	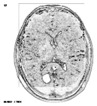	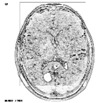	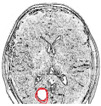
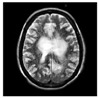	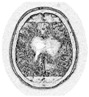	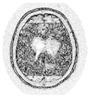	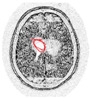
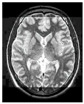	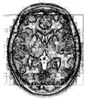	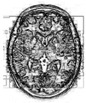	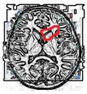
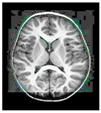	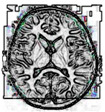	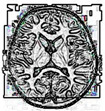	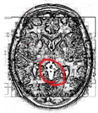

**Table 3:** Brain Tumor Segmentation Efficiency.

Methods	Accuracy	Specificity	Precision	Recall	F1 Score
DC-FOF [16]	0.856	0.834	0.86	0.893	0.892
HEC [18]	0.89	0.893	0.902	0.93	0.932
RBFNN [19]	0.902	0.902	0.91	0.945	0.95
MENF [21]	0.923	0.91	0.923	0.954	0.967
RG-FCM with HHOCNN (Proposed)	0.98	0.983	0.979	0.987	0.988

## Data Availability

Mentioned in the article’s Section 3.2.

## Appendix A

Source code for Feature Extraction

features=[];	
	
	i = imread(‘img.jpg’);
	subplot(1,3,1)
	imshow(i);
	title(‘Original Image’);
	
	gray=rgb2gray(i);
	subplot(1,3,2)
	imshow(gray);
	title(‘Converted to Grayscale image’);
	
	glcm=graycomatrix(gray)
	
	A=glcm;
	num_rows=size(A,1)
	num_cols=size(A,2)
	
	for i=1:num_rows
	for j=1:num_cols
	trans(i,j)=A(j,i);
	End
	End
	
	for i=1:1:num_rows
	for j=1:1:num_cols
	A(i,j)=A(i,j)+trans(i,j);
	End
	End
	
	factor=sum(sum(A));
	
	for i=1:1:num_rows
	for j=1:1:num_cols
	A(i,j)=A(i,j)/factor;
	End
	End
	
	disp(A);
	
	energy=0;
	for i=1:1:num_rows
	for j=1:1:num_cols
	energy = energy + A(i,j)^2;
	End
	End
	disp(energy);
	
	%Second Contrast
	
	contrast=0;
	for i=1:1:num_rows
	for j=1:1:num_cols
	contrast = contrast + A(i,j).*((i-j)^2);
	End
	End
	disp(contrast);
	
	%Third Homogeneity
	
	homogeneity=0;
	for i=1:1:num_rows
	for j=1:1:num_cols
	homogeneity = homogeneity + A(i,j).*(1/(1+(i-j)^2));
	End
	End
	disp(homogeneity);
	
	%Fourth Entropy
	for i=1:1:num_rows
	for j=1:1:num_cols
	
	B(i,j)=log(A(i,j));
	End
	End
	
	entropy=0;
	for i=1:1:num_rows
	for j=1:1:num_cols
	entropy = entropy - ( A(i,j)*B(i,j) );
	End
	End
	disp(entropy);
	
	features=[energy,contrast,homogeneity,entropy]
	
	x=0:0.33:1;
	
	subplot(1,3,3)
	plot(x,features,’r--s’)
	xlabel(‘X’)
	ylabel(‘Texture Features Values’)
	title(‘Plot of the energy,contrast,homogeneity,entropy’)

## Appendix B

	load Trainset
	
	% parameter setting
	alpha = 0.1; % learning rate
	beta = 0.01; % scaling factor for sigmoid function
	train_size = size(train_set);
	N = train_size(1); % number of training samples
	D = train_size(2); % dimension of feature vector
	n_hidden = 300; % number of hidden layer units
	K = 10; % number of output layer units
	% initialize all weights between -1 and 1
	W1 = 2*rand(1+D, n_hidden)-1; % weight matrix from input layer to hidden layer
	W2 = 2*rand(1+n_hidden, K)-1; % weight matrix from hidden layer to ouput layer
	max_iter = 100; % number of iterations
	Y = eye(K); % output vector
	
	% training
	for i=1:max_iter
	disp([num2str(i), ‘ iteration’]);
	for j=1:N
	% propagate the input forward through the network
	input_x = [1; train_set(j, :)’];
	hidden_output = [1;sigmf(W1’*input_x, [beta 0])];
	output = sigmf(W2’*hidden_output, [beta 0]);
	% propagate the error backward through the network
	% compute the error of output unit c
	delta_c = (output-Y(:,train_label(j)+1)).*output.*(1-output);
	% compute the error of hidden unit h
	delta_h = (W2*delta_c).*(hidden_output).*(1-hidden_output);
	delta_h = delta_h(2:end);
	% update weight matrix
	W1 = W1 - alpha*(input_x*delta_h’);
	W2 = W2 - alpha*(hidden_output*delta_c’);
	End
	End
	
	% testing
	test_size = size(test_set);
	num_correct = 0;
	for i=1:test_size(1)
	input_x = [1; test_set(i,:)’];
	hidden_output = [1; sigmf(W1’*input_x, [beta 0])];
	output = sigmf(W2’*hidden_output, [beta 0]);
	[max_unit, max_idx] = max(output);
	if(max_idx == test_label(i)+1)
	num_correct = num_correct + 1;
	End
	End
	% computing accuracy
	accuracy = num_correct/test_size(1);
